# Predictors of Nocturnal Hypoxemic Burden in Patients Undergoing Elective Coronary Artery Bypass Grafting Surgery

**DOI:** 10.3390/biomedicines11102665

**Published:** 2023-09-28

**Authors:** Maria Tafelmeier, Verka-Georgieva Blagoeva, Maximilian Trum, Philipp Hegner, Bernhard Floerchinger, Daniele Camboni, Marcus Creutzenberg, Florian Zeman, Christof Schmid, Lars Siegfried Maier, Stefan Wagner, Dominik Linz, Mathias Baumert, Michael Arzt

**Affiliations:** 1 Department of Internal Medicine II (Cardiology, Pneumology, and Intensive Care), University Medical Center Regensburg, 93053 Regensburg, Germany; verka-georgieva.blagoeva@stud.uni-regensburg.de (V.-G.B.); maximilian.trum@ukr.de (M.T.); philipp.hegner@ukr.de (P.H.); lars.maier@ukr.de (L.S.M.); stefan.wagner@ukr.de (S.W.); michael.arzt@ukr.de (M.A.); 2 Department of Cardiothoracic Surgery, University Medical Center Regensburg, 93053 Regensburg, Germany; bernhard.floerchinger@ukr.de (B.F.); daniele.camboni@ukr.de (D.C.); christof.schmid@ukr.de (C.S.); 3 Department of Anesthesiology, University Medical Center Regensburg, 93053 Regensburg, Germany; marcus.creutzenberg@ukr.de; 4 Center for Clinical Studies, University Medical Center Regensburg, 93053 Regensburg, Germany; florian.zeman@ukr.de; 5 Department of Cardiology, Maastricht University Medical Centre, 6229 ER Maastricht, The Netherlands; dominik.linz@mumc.nl; 6 Discipline of Biomedical Engineering, School of Electrical and Mechanical Engineering, The University of Adelaide, Adelaide, SA 5005, Australia; mathias.baumert@adelaide.edu.au

**Keywords:** T90, nocturnal hypoxemia, sleep apnea, heart failure

## Abstract

**Background:** Nocturnal hypoxemia has been linked to increased cardiovascular morbidity and mortality. Several common diseases, such as sleep-disordered breathing (SDB), heart failure (HF), obesity, and pulmonary disease, coincide with an elevated nocturnal hypoxemic burden with and without repetitive desaturations. **Research question:** This study aimed to evaluate the association of relevant common diseases with distinctive metrics of nocturnal hypoxemic burden with and without repetitive desaturations in patients undergoing coronary artery bypass grafting surgery. **Study design and methods:** In this subanalysis of the prospective observational study, CONSIDER-AF (NCT02877745) portable SDB monitoring was performed on 429 patients with severe coronary artery disease the night before cardiac surgery. Pulse oximetry was used to determine nocturnal hypoxemic burden, as defined by total recording time spent with oxygen saturation levels < 90% (T90). T90 was further characterized as T90 due to intermittent hypoxemia (T90_desaturation_) and T90 due to nonspecific and noncyclic SpO_2_-drifts (T90_non-specific_). **Results:** Multivariable linear regression analysis identified SDB (apnea–hypopnea-index ≥ 15/h; B [95% CI]: 6.5 [0.4; 12.5], *p* = 0.036), obesity (8.2 [2.5; 13.9], *p* = 0.005), and mild-to-moderate chronic obstructive pulmonary disease (COPD, 16.7 [8.5; 25.0], *p* < 0.001) as significant predictors of an increased nocturnal hypoxemic burden. Diseases such as SDB, obesity and HF were significantly associated with elevated T90_desaturation_. In contrast, obesity and mild-to-moderate COPD were significant modulators of T90_non-specific_. **Interpretation:** SDB and leading causes for SDB, such as obesity and HF, are associated with an increased nocturnal hypoxemic burden with repetitive desaturations. Potential causes for hypoventilation syndromes, such as obesity and mild-to-moderate COPD, are linked to an increased hypoxemic burden without repetitive desaturations. **Clinical Trial Registration:** ClinicalTrials.gov identifier: NCT02877745.

## 1. Background

Nocturnal hypoxemic burden is commonly defined as the total recording time (TRT) a patient spends at oxygen saturation levels below 90% (T90) [1,2]. More precisely, nocturnal hypoxemic burden may be attributed to both episodic oxygen desaturation–resaturation events (T90_desaturation_) and nonspecific transient drifts in oxygen saturation or incomplete resaturation (T90_non-specifc_) [3].

Although these metrics of nocturnal hypoxemic burden can easily be obtained by low-cost overnight oximetry, simpler measures of event frequency, such as the apnea–hypopnea index (AHI), or desaturation frequency, such as the oxygen desaturation index (ODI), are more conventionally used in daily clinical practice. However, these frequency-based measures fail to incorporate the depth and duration of oxygen desaturations and thus inadequately reflect the physiological disturbances that may ultimately have a detrimental impact on clinical outcomes [4].

Metrics of nocturnal hypoxemic burden have recently been linked to adverse clinical outcomes [3,5]. In contrast to AHI, nocturnal hypoxemic burden was independently associated with cardiovascular mortality and all-cause mortality in two large samples of middle-aged and older adults from several communities in the USA [5]. Moreover, nocturnal hypoxemic burden was found to be an independent predictor of cardiovascular mortality in community-dwelling older men, and both components of nocturnal hypoxemic burden (i.e., T90_desaturation_ and T90_non-specific_) contributed towards the association with cardiovascular mortality [3].

To date, nocturnal hypoxemic burden has mainly been examined in sleep-disordered breathing (SDB), when obstructive events cause intermittent hypoxemia. Besides, persistent hypoxemia could also be a consequence of common diseases, such as heart failure (HF), obesity, and pulmonary disease and may also contribute to an elevated nocturnal hypoxemic burden with and without repetitive desaturations [6].

The objective of the present study was to evaluate the association of relevant common diseases with distinctive metrics of nocturnal hypoxemic burden with and without repetitive desaturations in patients with severe coronary artery disease undergoing elective coronary artery bypass grafting surgery. Hence, the results of this study may contribute to deeper insights into specific risk clusters for severe nocturnal hypoxemia.

## 2. Methods

### 2.1. Study Design and Patients

The present subanalysis is part of the ongoing prospective observational study ‘Impact of Sleep-disordered breathing on Atrial Fibrillation and Perioperative complications in patients with severe coronary artery disease undergoing Coronary Artery Bypass grafting Surgery’ (CONSIDER AF, NCT02877745) that assesses the impact of SDB on the rate of Major Adverse Cardiac and Cerebrovascular Events in patients undergoing elective coronary artery bypass grafting (CABG) surgery at the Department of Cardiothoracic Surgery of the University Medical Center Regensburg Germany [7]. This study was approved by the Ethics Committee of the University of Regensburg (no. 15-101-0238).

Between May 2016 and June 2021, elective patients aged between 18 and 85 years were tested for eligibility. Informed consent was obtained from all eligible patients willing to participate in this study. As outlined beforehand [7], the exclusion criteria were severe chronic obstructive pulmonary disease, oxygen therapy, nocturnal positive airway pressure support or mechanical ventilation, and the need for catecholamines or circulatory assist devices [7].

### 2.2. SDB Monitoring

The night before CABG, portable SDB monitoring was performed using the Alice NightOne device (Philips Respironics, Murrysville, PA, USA) with three sensors that measure nasal flow, pulse oximetry, and thoracic breathing effort [7]. The data acquired by the Alice NightOne devices were systematically scored by trained medical staff using the corresponding Sleepware G3 sleep diagnostic software (Philips Respironics, Murrysville, PA, USA).

As described previously [7], apnea was defined as a ≥90% decrease in airflow for ≥10 s, hypopnea as a decrease in airflow by ≥30–90% versus baseline for ≥10 s, and desaturation as a ≥4% decrease in oxygen saturation [8,9]. The apnea–hypopnea index (AHI) is expressed as the frequency of apnea or hypopnea events per hour recording time. An AHI of ≥15/h was considered the cut-off for diagnosing SDB. Moreover, apneas and hypopneas were scored as either obstructive or central events, according to the American Academy of Sleep Medicine [9]. In brief, obstructive apnea events involved a ≥90% decrease in airflow despite an ongoing effort to breathe, whereas during central apnea events, there was a lack of ventilatory effort or drive to breathe. Patients with SDB and ≥50% of central apnea events were classified into the central sleep apnea (CSA) group, and patients with <50% of central apnea events into the obstructive sleep apnea (OSA) group.

### 2.3. Quantification and Characterization of Nocturnal Hypoxemia

Oximetry signals were extracted from the SDB-monitoring data for further processing using a novel, fully automated, and custom-made computer algorithm programmed in MATLAB^®^ (MathWorks^®^, Natick, MA, USA), as described previously [6]. Signal artifacts were automatically detected and excluded based on empirical criteria (e.g., instantaneous changes in SpO_2_ > 5%).

Nocturnal hypoxemic burden (T90) and nocturnal hypoxemic burden index (T90/TRT) were defined as artifact-free total recording time (TRT) and percentage of TRT spent at SpO_2_ levels below 90%, respectively [6]. To further characterize the composition of nocturnal hypoxemic burden, we differentiated the component of T90 associated with acute oxygen desaturation events accompanied by resaturation (T90_desaturation_) versus T90 associated with nonspecific and noncyclic drifts in SpO_2_ or incomplete resaturation (T90_non-specific_) [6]. Please see Figure S1 for a schematic example of T90_desaturation_ and T90_non-specific_.

### 2.4. Stratification of Patients by Relevant Diseases

For analyses of baseline characteristics and respiratory data, patients were stratified into seven subgroups by the presence, absence, and concomitance of certain diseases potentially predisposing to hypoxemia, such as heart failure, mild-to-moderate COPD, obesity, and SDB. Diagnosis of heart failure was based on the patient’s medical records and age-stratified cut-off levels of NT-proBNP (patients < 50 years of age: ≥450 pg/mL; patients ≥ 50 and <75 years of age: ≥900 pg/mL; patients ≥ 75 years of age: ≥1800 pg/mL) [10,11].

Mild-to-moderate COPD was diagnosed through a combination of the patient’s medical history and spirometry data with a forced expiratory volume/forced vital capacity (FEV_1_/FVC) < 0.70, confirming persistent airflow limitation [12]. Patients were classified into Global Initiative for Chronic Obstructive Lung Disease (GOLD) categories 1 and 2 according to their level of airflow limitation severity as assessed by FEV_1_-values [12]. Owing to the prespecified exclusion criteria of CONSIDER-AF, patients with GOLD categories 3 and 4 were excluded from the present study. Obesity was defined by a body mass index of ≥30 kg/m^2^ [13].

### 2.5. Data Management and Statistical Analysis

Data management and statistical analysis were conducted according to the data handling plan described in the published study protocol of the CONSIDER-AF study [7]. Statistical analyses were performed with SPSS 26.0 (IBM, New York, NY, USA). Data are presented as mean ± standard deviation for normally distributed data and as median (25.;75. percentile) for non-normally distributed data; categorical variables are described as absolute and relative frequencies. Differences between groups were compared using the Student’s *t*-test or the ANOVA test for normally distributed continuous variables, the Mann-Whitney-U test or the Kruskal-Wallis test for non-normally distributed continuous variables, and the Pearson’s chi-square test of independence for categorical variables. Univariable linear regression analyses were conducted with potential predictors for an increased nocturnal hypoxemic burden index, namely heart failure, mild-to-moderate COPD, obesity, and SDB (AHI ≥ 15/h) as independent variables and with three metrics of an increased nocturnal hypoxemic burden index, namely T90/TRT, T90_desaturation_/TRT, or T90_non-specific_/TRT, as dependent variables. Multivariable linear regression models for T90/TRT, T90_desaturation_/TRT, or T90_non-specific_/TRT as dependent variables were calculated that were adjusted for key demographic parameters (model I) and, additionally, all independent variables (model II), as mentioned beforehand. Beta coefficient (B) and 95%-confidence intervals (CI) are presented as effect estimates. A two-sided *p*-value of ≤0.05 was considered statistically significant for all analyses.

## 3. Results

### 3.1. Study Patients

**Figure 1:**
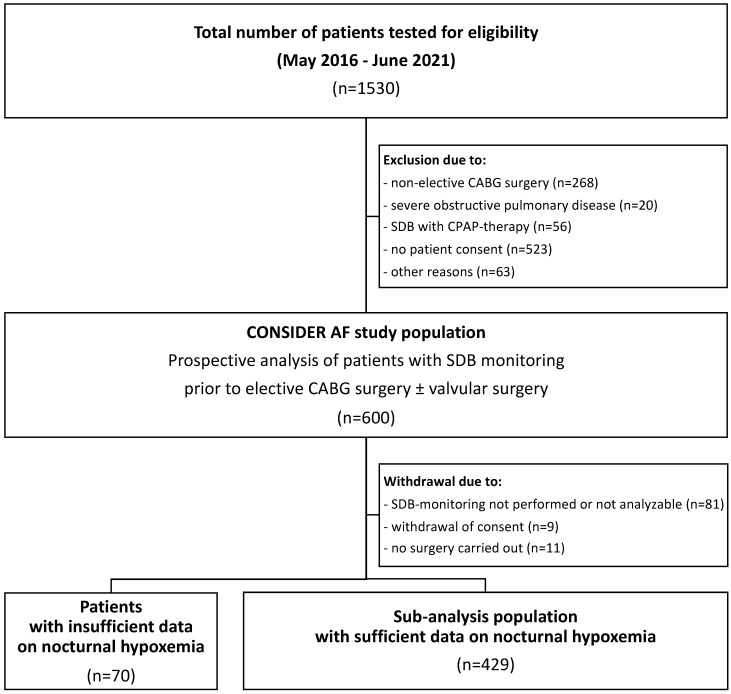
Study flowchart. CABG: coronary artery bypass grafting; SDB: sleep-disordered breathing; CPAP: continuous positive airway pressure.

Between May 2016 and June 2021, 600 patients were recruited for the ongoing prospective observational study CONSIDER-AF. Preponderantly due to unperformed or unanalyzable SDB monitoring, withdrawal of consent, or short-term cancellation of CABG surgery, 101 patients were excluded from the CONSIDER-AF cohort (Figure 1). Seventy patients were omitted from the present subanalysis of CONSIDER-AF due to insufficient data on nocturnal hypoxemia. Thus, the final subanalysis cohort consisted of 429 patients who were classified according to their nocturnal hypoxemic burden (Figure 1). The demographics of patients who were excluded from the present subanalysis or had insufficient data on nocturnal hypoxemia were similar to the sub-analysis cohort (Tables S1 and S2).

Most participants were elderly and overweight men (Table 1). The prevalence of cardiovascular risk factors and other comorbidities, as well as echocardiographic parameters, laboratory, and preoperative data, are summarized in Table 1. Previously undiagnosed SDB (AHI ≥ 15/h) was present in 46% of all patients, comprising 19% OSA and 27% CSA (Table 2). Nocturnal respiration data are summarized in Table 2. Baseline and nocturnal respiration data stratified by the presence or absence of common comorbidities (i.e., heart failure, mild-to-moderate COPD, obesity, and SDB) are shown in Tables S3 and S4, respectively.

### 3.2. Nocturnal Hypoxemic Burden in Distinctive Comorbidities

**Figure 2:**
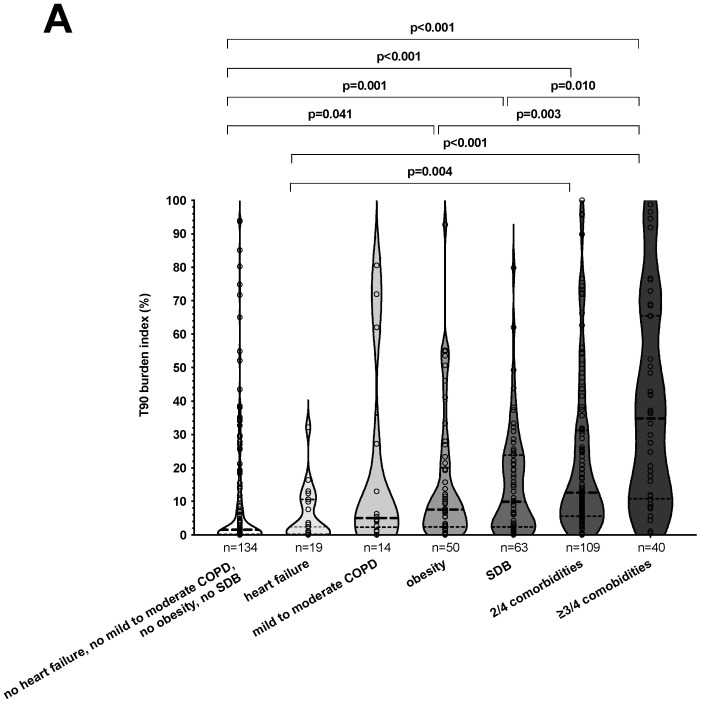

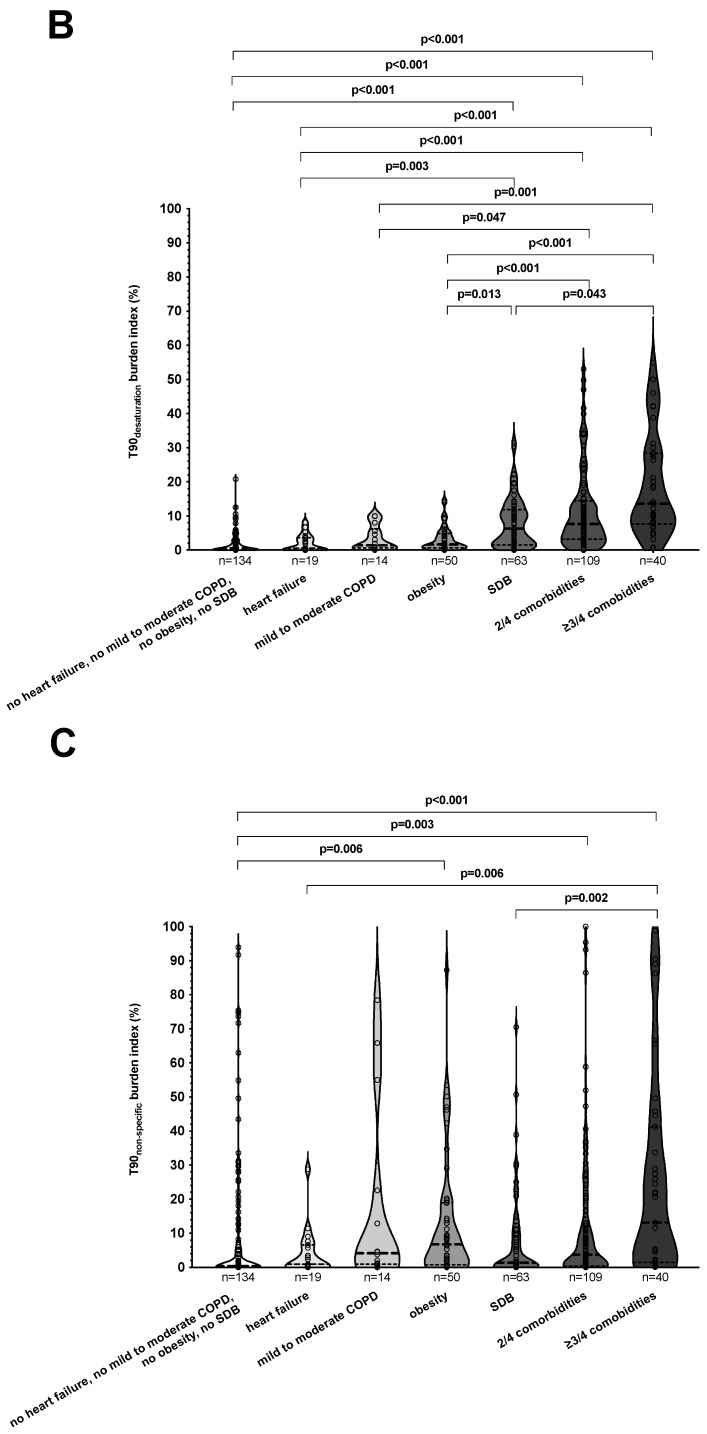
Nocturnal hypoxemic burden index according to different comorbidities. The violin plots indicate the median and quartiles of the nocturnal hypoxemic burden index in percent of artifact-free total recording time according to different combinations of comorbidities for T90/TRT (**A**), T90_desaturation_/TRT (**B**), and T90_non-specific_/TRT (**C**). The violin plot outlines illustrate kernel probability density, i.e., the width of the shaded area represents the proportion of the data. SDB: sleep-disordered breathing; COPD: chronic obstructive pulmonary disease.

Median values for T90/TRT, T90_desaturation_/TRT, and T90_non-specific_/TRT were 8.0% (1.3; 25.2), 3.1% (0.6; 9.6), and 2.2% (0.1; 13.3) for all patients, respectively. In the absence of relevant comorbidities, such as heart failure, mild-to-moderate COPD, obesity, and SDB, median values for T90/TRT (Figure 2A), T90_desaturation_/TRT (Figure 2B), and T90_non-specific_/TRT (Figure 2C) were the lowest. Depending on the concomitance of multiple comorbidities, median values for T90/TRT (Figure 2A), T90_desaturation_/TRT (Figure 2B), and T90_non-specific_/TRT (Figure 2C) varied significantly. Median T90/TRT was the highest in patients with a concurrent diagnosis of at least three out of four comorbidities (34.8% [10.8; 65.5]; Figure 2A). The same was true for median T90_desaturation_/TRT and median T90_non-specific_/TRT. The highest median values for T90_desaturation_/TRT (13.6% [7.7; 28.4]; Figure 2B) and median T90_non-specific_/TRT (13.1% [1.4; 41.3]; Figure 2C) were detected in patients suffering from at least three out of four comorbidities. Please refer to Figure 2 for significant pairwise comparisons.

### 3.3. Association of Different Diseases with an Increased Nocturnal Hypoxemic Burden

**Figure 3:**
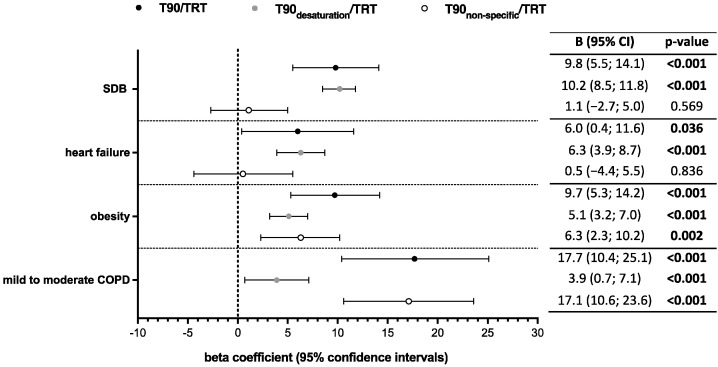
Association of different comorbidities with an increased nocturnal hypoxemic burden index. Forest plot depicting the association between different comorbidities and metrics of an increased nocturnal hypoxemic burden index (i.e., T90/TRT, T90_desaturation_/TRT, and T90_non-specific_/TRT). Values are presented as B: beta coefficient and 95% CI: confidence interval. SDB: sleep-disordered breathing; TRT: artifact-free total recording time; COPD: chronic obstructive pulmonary disease.

In univariable linear regression analyses, SDB, heart failure, obesity, and mild-to-moderate COPD were significantly associated with both an increased T90/TRT and an increased T90_desaturation_/TRT (Figure 3). In contrast, only obesity and mild-to-moderate COPD were significant predictors of an increased T90_non-specific_/TRT in univariable linear regression analysis (Figure 3).

After adjusting for age, sex, and all independent variables using multivariable linear regression, SDB, obesity, and mild-to-moderate COPD remained significantly associated with an increased T90/TRT, whereas heart failure was no longer statistically significant in the fully adjusted model (Table 3). Alongside SDB, heart failure, obesity, and mild-to-moderate COPD were identified as independent modulators for an increased T90_desaturation_/TRT (Table 3). Applying multivariable linear regression, the association between obesity and mild-to-moderate COPD with an increased T90_non-specific_/TRT remained statistically significant (Table 3).

## 4. Discussion

This subanalysis of CONSIDER-AF provides novel insights into the association of relevant diseases with nocturnal hypoxemia with and without repetitive desaturations in patients undergoing elective coronary artery bypass grafting surgery. First, median values for T90, T90_desat_, and T90_non-specific_ vary significantly depending on the concomitance of multiple diseases, such as heart failure, mild-to-moderate COPD, obesity, and SDB. Second, SDB and leading causes for SDB, such as obesity and HF, are associated with an increased nocturnal hypoxemic burden with repetitive desaturations (T90_desat_). Third, potential causes for hypoventilation syndromes, such as obesity and mild-to-moderate COPD, are linked to an increased nonspecific nocturnal hypoxemic burden (T90_non-specific_).

### 4.1. Nocturnal Hypoxemia in Various Comorbidities

In our study, median T90/TRT was significantly associated with SDB, heart failure, obesity, and COPD. Remarkably, multiple, co-existing comorbidities contributed to an even further increased nocturnal hypoxemic burden, with the highest T90/TRT values being reported for patients with a concurrent diagnosis of at least three out of four comorbidities. As T90_desat_ represents desaturations due to acute obstructions, median T90_desat_ was significantly associated with SDB and comorbidities closely associated with SDB, such as heart failure and obesity.

Due to a bidirectional relationship, CSA is often regarded as a sign of unstable respiratory control in patients with heart failure [14]. Briefly, an overload of fluid in heart failure, followed by a night-time shift of fluid towards the upper body, leads to a constriction of the upper airway and an unsteady control of breathing [15,16]. As a result, the nocturnal rostral fluid shift amplifies the severity of both OSA and CSA [16,17]. Conversely, sleep apnea can exacerbate heart failure by exposing the heart to intermittent hypoxia, increased preload and afterload, augmented sympathetic drive, and vascular endothelial impairment [18]. The bidirectional relationship between sleep apnea and heart failure is depicted in Figure S2 (modified after Parati G et al. 2016 [2]).

The association between nocturnal hypoxemia and incident heart failure was assessed using the data from the SHHS and MrOS studies [4,19]. The incidence of heart failure was significantly higher in male patients with high hypoxemic burden regardless of the AHI, which underlines the importance of considering the duration and depth of the desaturation and their relation to heart failure prediction [4,19]. Computational simulation shows that there is a strong interaction between the epicardial blood flow and distal microcirculatory resistance [20]. The hemodynamic changes induced by the proximal graft could trigger changes in microcirculation that involve interactions between the proximal and distal circulations on both hemodynamic and metabolic levels. Such interactions could be associated with problems of cardiorespiratory regulation and merit further investigation.

As our data implies, nocturnal hypoxemia that is predominantly caused by repetitive desaturations (i.e., T90_desat_) may be sufficiently addressed by positive airway pressure therapy.

Worldwide, obesity has nearly tripled over the last four decades, and, to date, approximately 39% of adults are considered overweight, and 13% are classified as obese [21]. Obesity hypoventilation syndrome (OHS) is a common disorder in morbidly obese patients that is characterized by alveolar hypoventilation during sleep and wakefulness [22]. OHS is caused by a complex interaction between impaired respiratory mechanics due to central fat accumulation, decreased ventilatory drive secondary to blunted neural response to hypercapnia, and sleep-disordered breathing [22]. Obesity was significantly associated with both T90_desat_ as well as T90_non-specific_. Early diagnosis and the initiation of proper treatment are crucial, as hypoxemia is an independent predictor of mortality in OHS [23,24]. Continuous positive airway pressure is generally considered the first-line treatment modality for OHS with co-existing severe obstructive sleep apnea, whereas noninvasive ventilation is favored in the minority of OHS patients with hypoventilation during sleep with no or milder forms of OSA [25]. In addition, weight loss and bariatric surgery were shown to be effective in improving nocturnal hypoxemia in obese patients [26].

Nocturnal hypoxemia frequently occurs in patients with COPD, with prevalence estimates ranging between 25% and 50% [27,28,29]. In particular, patients with severe forms of COPD and patients with COPD and concomitant SDB (i.e., overlap syndrome) are prone to experience pronounced nocturnal hypoxemia [30]. Pathophysiologically, hypoxemia in patients with COPD is mainly due to ventilation/perfusion mismatch resulting from progressive airflow limitation and emphysematous destruction of the pulmonary capillary bed, which may be exacerbated during sleep [30]. As diaphragmatic efficiency is reduced because of pulmonary hyperinflation, patients with COPD rely on the contraction of their accessory respiratory muscles, which is markedly reduced during rapid-eye-movement sleep and, thus, contributes to nocturnal hypoventilation and hypoxemia [30]. Hypoxemia in patients with COPD has been linked to several harmful sequelae, such as pulmonary hypertension, secondary polycythemia, systemic inflammation, and skeletal muscle dysfunction, that may ultimately lead to reduced exercise tolerance, diminished quality of life, increased risk of cardiovascular morbidity, and greater risk of death [30]. Nonspecific nocturnal hypoxemia (i.e., T90_non-specific_) in patients with COPD may be treated by long-term oxygen therapy, which has been shown to improve pulmonary hemodynamics, reduce erythrocytosis, and improve survival in patients with severe hypoxemic respiratory failure [30], especially in the case of complications, such as pulmonary hypertension or polycythemia.

### 4.2. Strengths and Limitations

All components of nocturnal hypoxemia were autoscored using a custom-made automated algorithm that has previously been used and validated [3,6] without further manual adjustments by specialists. Manual scoring is generally considered time-consuming and tedious, with noticeable interrater variability [31]. In contrast, autoscoring of T90, T90_desat_, and T90_non-specific_ using a computer algorithm offers valuable additional information on nocturnal hypoxemia with and without repetitive desaturations that is readily available and could be easily transferred to routine clinical scoring practice.

The neural regulation of the cardiovascular–respiratory system was reported to influence the baseline and frequency [32] as well as the waveform [33] of photoplethysmography signals of pulse oximeters. Nowadays, pulse oximeters are commonplace on wearables, such as smartwatches and fitness trackers, that become increasingly popular as health monitoring devices. The results of our study may serve as a basis for future investigations on pathophysiological mechanisms that may ultimately improve machine-learning algorithms in wearables.

This study has some limitations. Our data were obtained within a typical cardiac surgery cohort with high proportions of patients with concomitant SDB and/or HF [34]. In line with current data on men and women presenting for CABG, our patient cohort comprises predominantly men [35]. Moreover, our findings may be affected by selection bias since patients with severe or very severe COPD (as defined by GOLD stage III or IV) were excluded from participating in our prospective observational study [7]. Thus, our findings may not be generalized to women or other patient populations.

## 5. Conclusions

In conclusion, our study details the extent to which relevant diseases contribute to nocturnal hypoxemia with and without repetitive desaturations. The results of this study support the concept that assessment of hypoxemic burden decomposed into its desaturation-related components and nonspecific drifts may contribute to the identification of specific risk clusters, including relevant diseases predisposing to a specific phenotype of hypoxia. In addition, as therapeutic approaches vary depending on the predominant type of nocturnal hypoxemia (i.e., T90_desat_ or T90_non-specific_), our data may serve as a basis for future patient-tailored treatment strategies to reduce nocturnal hypoxemic burden.

## Figures and Tables

**Table 1:** Patient characteristics.

	Total Subanalysis Population
Demographic data	
n (%)	429 (100)
Age, years	66.6 ± 8.5
Male sex, n (%)	368 (86)
Body mass index, kg/m^2^	28.6 ± 4.4
Cardiovascular risk factors	
Hypertension, n (%)	356 (83)
Hypercholesterinemia, n (%)	266 (62)
Diabetes mellitus, n (%)	142 (33)
Obesity, n (%)	157 (37)
Smoking, n (%)	288 (67)
Comorbidities	
Heart failure *, n (%)	87 (20)
NYHA III/IV, n (%)	96 (22)
History of myocardial infarction, n (%)	122 (29)
Atrial fibrillation, n (%)	60 (14)
History of transient ischemic attack or stroke, n (%)	58 (13)
Mild-to-moderate chronic obstructive pulmonary disease, n (%)	44 (10)
Renal failure ^†^, n (%)	88 (21)
Anemia ^‡^, n (%)	87 (20)
Depression, n (%)	19 (4)
Echocardiography parameters	
LV ejection fraction, %	55.3 ± 11.7
Impaired LV ejection fraction < 55%, n (%)	98 (29)
Left atrial enlargement, n (%)	154 (48)
Laboratory data	
NT-proBNP, pg/mL	315 (104; 906)
Hemoglobin, g/dL	14.1 (13.1; 15.0)
Hb1Ac, g/dL	5.9 (5.5; 6.4)
Creatinine, mg/dL	0.97 (0.84; 1.12)
GFR, mL/min/1.73 m^2^	80 (64; 90)
Preoperative information on surgical treatment	
CABG and valve surgery, n (%)	95 (22)
Number of coronary stenoses, n	3 (3; 5)
Number of grafts, n	2 (2; 3)

Data are presented as mean ± standard deviation, median (interquartile range) or absolute and relative frequencies. NYHA: New York Heart Association; LV: left ventricular; NT-proBNP: N-terminal probrain natriuretic peptide; HbA1c: glycosylated Hemoglobin, Type A1C; GFR: glomerular filtration rate; CABG: coronary artery bypass grafting. * n = 363; NT-proBNP ≥ 450 pg/mL (patients < 50 years of age), ≥900 pg/mL (patients ≥ 50 and <75 years of age) or ≥1800 pg/mL (patients ≥ 75 years of age); ^†^ glomerular filtration rate < 60 mL/min/1.73 m^2^; ^‡^ hemoglobin < 12 g/dL (women) or hemoglobin <13 g/dL (men).

**Table 2:** Metrics of nocturnal respiration.

	Total Subanalysis Population
Total recording time, min	429 (405; 448)
Apnea–hypopnea index, per hour of recording	14.1 (6.9; 24.2)
Obstructive apnea index, per hour of recording	2.0 (0.8; 4.9)
Central apnea index, per hour of recording	2.0 (0.5; 6.9)
Oxygen desaturation index, per hour of recording	10.4 (4.4; 20.9)
Mean SpO_2_, %	92 (91; 93)
Min SpO_2_, %	83 (78; 86)
Sleep-disordered breathing (Apnea–hypopnea index ≥ 15/h), n (%)	198 (46)
Obstructive sleep apnea (Apnea–hypopnea index ≥ 15/h), n (%)	82 (19)
Central sleep apnea (Apnea–hypopnea index ≥ 15/h), n (%)	116 (27)

Data are presented as median (interquartile range) or absolute and relative frequencies. TRT: artifact-free total recording time.

**Table 3:** Association of different comorbidities with an increased nocturnal hypoxemic burden using multivariable linear regression.

		Adjusted for Age and Sex		Adjusted for Age, Sex, and All Independent Variables	
		B (95% CI)	*p*-Value	B (95% CI)	*p*-Value
Dependent Variable: T90/TRT					
independent variables	Sleep-disordered breathing (AHI ≥ 15/hour)	9.7 (5.4; 14.0)	<0.001	6.5 (0.4; 12.5)	0.036
	Heart failure	6.3 (0.7; 11.8)	0.027	3.8 (−3.0; 10.6)	0.273
	Obesity	9.9 (5.4; 14.3)	<0.001	8.2 (2.5; 13.9)	0.005
	Mild-to-moderate chronic obstructive pulmonary disease	18.6 (11.3; 25.9)	<0.001	16.7 (8.5; 25.0)	<0.001
Dependent Variable: T90_desaturation_/TRT					
independent variables	Sleep-disordered breathing (AHI ≥ 15/hour)	10.0 (8.3; 11.6)	<0.001	8.4 (6.1; 10.7)	<0.001
	Heart failure	6.3 (4.0; 8.7)	<0.001	3.7 (1.1; 6.4)	0.006
	Obesity	5.3 (3.4; 7.2)	<0.001	2.9 (0.7; 5.1)	0.010
	Mild-to-moderate chronic obstructive pulmonary disease	4.3 (1.1; 7.5)	0.008	3.6 (0.4; 6.8)	0.026
Dependent Variable: T90_non-specific_/TRT					
independent variables	Sleep-disordered breathing (AHI ≥ 15/hour)	1.1 (−2.7; 5.0)	0.559	−1 (−6.6; 4.5)	0.712
	Heart failure	0.8 (−4.1; 5.7)	0.756	0.05 (−6.2; 6.3)	0.988
	Obesity	6.3 (2.3; 10.2)	0.002	7.1 (1.9; 12.2)	0.008
	Mild-to-moderate chronic obstructive pulmonary disease	17.7 (11.2; 24.2)	<0.001	17 (9.5; 24.6)	<0.001

B: beta coefficient and 95% CI: confidence interval. AHI: apnea–hypopnea index; TRT: artifact-free total recording time.

## Data Availability

The work was submitted to the European Respiratory Society meeting on 9/2023 in Milan, Italy.

## Supplementary Materials

The following supporting information can be downloaded: https://www.mdpi.com/article/10.3390/biomedicines11102665/s1, Table S1. Baseline variables of the sub-analysis population and patients excluded from the sub-analysis or had insufficient data on nocturnal hypoxemia (drop-out population). Data are presented as absolute and relative frequencies or mean ± standard deviation. Table S2. Baseline variables of the sub-analysis population and patients excluded from the sub-analysis or had insufficient data on nocturnal hypoxemia. Data are presented as absolute and relative frequencies or mean ± standard deviation. Table S3. Patient characteristics. Data are presented as mean ± standard deviation, median (interquartile range) or absolute and relative frequencies. Table S4. Nocturnal respiration data of the study population of patients (n = 429) without and with an elevated nocturnal hypoxemic burden index. Data are presented as median (interquartile range) or absolute and relative frequencies. Figure S1. Descriptive diagram of T90 with acute oxygen desaturation events accompanied by resaturation (T90desaturation) and T90 associated with non-specific drifts in SpO2 or incomplete resaturation (T90non-specific) as obtained from raw oximetry data with the use of a custom MATLAB software (Version 9.13) algorithm. Figure S2. Schematic model depicting the relationship between sleep apnea and heart failure (illustration modified from Parati G et al. 2016) [2]. RAA: Renin-angiotensin-aldosterone; VO2: oxygen consumption.

## Abbreviations

AHI	Apnea–hypopnea index
B	beta coefficient
CABG	coronary artery bypass grafting
CI	95%-confidence intervals
COPD	chronic obstructive pulmonary disease
CSA	central sleep apnea
FEV_1_/FVC	forced expiratory volume/forced vital capacity
GOLD	Global Initiative for Chronic Obstructive Lung Disease
HF	heart failure
ODI	oxygen desaturation index
OHS	obesity hypoventilation syndrome
OSA	obstructive sleep apnea
SDB	sleep-disordered breathing
T90	nocturnal hypoxemic burden (i.e., oxygen saturation levels < 90%)
T90/TRT	nocturnal hypoxemic burden index
T90_desaturation_	T90 due to intermittent hypoxemia
T90_non-specific_	T90 due to nonspecific and noncyclic SpO_2_-drifts
TRT	total recording time

## References

[1] Oldenburg O., Costanzo M.R., Germany R., McKane S., Meyer T.E., Fox H. (2021). Improving Nocturnal Hypoxemic Burden with Transvenous Phrenic Nerve Stimulation for the Treatment of Central Sleep Apnea. J. Cardiovasc. Transl. Res..

[2] Parati G., Lombardi C., Castagna F., Mattaliano P., Filardi P.P., Agostoni P., on behalf of the Italian Society of Cardiology (SIC) Working Group on Heart Failure members (2016). Heart failure and sleep disorders. Nat. Rev. Cardiol..

[3] Baumert M., A Immanuel S., Stone K.L., Harrison S.L., Redline S., Mariani S., Sanders P., McEvoy R.D., Linz D. (2020). Composition of nocturnal hypoxaemic burden and its prognostic value for cardiovascular mortality in older community-dwelling men. Eur. Heart J..

[4] Martinez-Garcia M.A., Sanchez-de-la-Torre M., White D.P., Azarbarzin A. (2023). Hypoxic Burden in Obstructive Sleep Apnea: Present and Future. Arch. Bronconeumol..

[5] Azarbarzin A., A Sands S., Stone K.L., Taranto-Montemurro L., Messineo L., I Terrill P., Ancoli-Israel S., Ensrud K., Purcell S., White D.P. (2019). The hypoxic burden of sleep apnoea predicts cardiovascular disease-related mortality: The Osteoporotic Fractures in Men Study and the Sleep Heart Health Study. Eur. Heart J..

[6] Linz D., Malfertheiner M.V., Werner N., Lerzer C., Gfüllner F., Linz B., Zeman F., McEvoy R.D., Arzt M., Baumert M. (2021). Nocturnal hypoxemic burden during positive airway pressure treatment across different central sleep apnea etiologies. Sleep Med..

[7] Tafelmeier M., Knapp M., Lebek S., Floerchinger B., Camboni D., Wittmann S., Creutzenberg M., Zeman F., Schmid C., Maier L.S. (2019). Rationale and design of the CONSIDER AF study: Impact of sleep-disordered breathing on atrial fibrillation and perioperative complications in patients undergoing coronary artery bypass grafting surgery. Somnologie.

[8] Berry R.B., Gamaldo C.E., Harding S.M., Brooks R., Lloyd R.M., Vaughn B.V., Marcus C.L. (2015). AASM Scoring Manual Version 2.2 Updates: New Chapters for Scoring Infant Sleep Staging and Home Sleep Apnea Testing. J. Clin. Sleep Med..

[9] Berry R.B., Brooks R., Gamaldo C., Harding S.M., Lloyd R.M., Quan S.F., Troester M.T., Vaughn B.V. (2017). AASM Scoring Manual Updates for 2017 (Version 2.4). J. Clin. Sleep Med..

[10] Januzzi J.L., Chen-Tournoux A.A., Christenson R.H., Doros G., Hollander J.E., Levy P.D., Nagurney J.T., Nowak R.M., Pang P.S., Patel D. (2018). N-Terminal Pro-B-Type Natriuretic Peptide in the Emergency Department: The ICON-RELOADED Study. J. Am. Coll. Cardiol..

[11] McCullough P.A., Kluger A.Y. (2018). Interpreting the Wide Range of NT-proBNP Concentrations in Clinical Decision Making. J. Am. Coll. Cardiol..

[12] Agustí A., Celli B.R., Criner G.J., Halpin D., Anzueto A., Barnes P., Bourbeau J., Han M.K., Martinez F.J., Montes de Oca M. (2023). Global Initiative for Chronic Obstructive Lung Disease 2023 Report: GOLD Executive Summary. Eur. Respir. J..

[13] Ortega F.B., Lavie C.J., Blair S.N. (2016). Obesity and Cardiovascular Disease. Circ. Res..

[14] Javaheri S. (2006). Sleep disorders in systolic heart failure: A prospective study of 100 male patients. The final report. Int. J. Cardiol..

[15] Chiu K.-L., Ryan C.M., Shiota S., Ruttanaumpawan P., Arzt M., Haight J.S., Chan C.T., Floras J.S., Bradley T.D. (2006). Fluid shift by lower body positive pressure increases pharyngeal resistance in healthy subjects. Am. J. Resp. Crit. Care.

[16] Yumino D., Redolfi S., Ruttanaumpawan P., Su M.C., Smith S., Newton G.E., Mak S., Bradley T.D. (2010). Nocturnal rostral fluid shift: A unifying concept for the pathogenesis of obstructive and central sleep apnea in men with heart failure. Circulation.

[17] Redolfi S., Yumino D., Ruttanaumpawan P., Yau B., Su M.-C., Lam J., Bradley T.D. (2009). Relationship between Overnight Rostral Fluid Shift and Obstructive Sleep Apnea in Nonobese Men. Am. J. Resp. Crit. Care.

[18] Buchner S., Greimel T., Hetzenecker A., Luchner A., Hamer O.W., Debl K., Poschenrieder F., Fellner C., Riegger G.A., Pfeifer M. (2012). Natural course of sleep-disordered breathing after acute myocardial infarction. Eur. Respir. J..

[19] Azarbarzin A., Sands S.A., Taranto-Montemurro L., Vena D., Sofer T., Kim S.-W., Stone K.L., White D.P., Wellman A., Redline S. (2020). The Sleep Apnea-Specific Hypoxic Burden Predicts Incident Heart Failure. Chest.

[20] Liu H., Ou S., Liu P., Xu Y., Gong Y., Xia L., Leng X., Leung T.W.H., Shi L., Zheng D. (2020). Effect of microcirculatory resistance on coronary blood flow and instantaneous wave-free ratio: A computational study. Comput. Methods Prog. Biomed..

[21] World Health Organization (2021). Obesity and Overweight. https://www.who.int/news-room/fact-sheets/detail/obesity-and-overweight.

[22] Shetty S., Parthasarathy S. (2015). Obesity Hypoventilation Syndrome. Curr. Pulmonol. Rep..

[23] Budweiser S., Riedl S.G., Jorres R.A., Heinemann F., Pfeifer M. (2007). Mortality and prognostic factors in patients with obesity-hypoventilation syndrome undergoing noninvasive ventilation. J. Intern. Med..

[24] Priou P., Hamel J.-F., Person C., Meslier N., Racineux J.-L., Urban T., Gagnadoux F. (2010). Long-term outcome of noninvasive positive pressure ventilation for obesity hypoventilation syndrome. Chest.

[25] Masa J.F., Pepin J.L., Borel J.C., Mokhlesi B., Murphy P.B., Sanchez-Quiroga M.A. (2019). Obesity hypoventilation syndrome. Eur. Respir. Rev..

[26] Zhang Y., Wang W., Yang C., Shen J., Shi M., Wang B. (2019). Improvement in Nocturnal Hypoxemia in Obese Patients with Obstructive Sleep Apnea after Bariatric Surgery: A Meta-Analysis. Obes. Surg..

[27] Lacasse Y., Sériès F., Vujovic-Zotovic N., Goldstein R., Bourbeau J., Lecours R., Aaron S.D., Maltais F. (2011). Evaluating nocturnal oxygen desaturation in COPD—Revised. Respir. Med..

[28] Lewis C.A., Fergusson W., Eaton T., Zeng I., Kolbe J. (2009). Isolated nocturnal desaturation in COPD: Prevalence and impact on quality of life and sleep. Thorax.

[29] Gupta P., Chhabra S. (2015). Prevalence, predictors and impact of nocturnal hypoxemia in non-apnoeic patients with COPD. Eur. Respir. J..

[30] Kent B.D., Mitchell P.D., McNicholas W.T. (2011). Hypoxemia in patients with COPD: Cause, effects, and disease progression. Int. J. Chron. Obs. Pulmon. Dis..

[31] Anderer P., Ross M., Cerny A., Moreau A. (2020). 0435 Autoscoring of Sleep and Associated Events Versus a Reference Scorer Competing with Three Additional Manual Scorings: A Clinical Validation Study. Sleep.

[32] Liu H., Chen F., Hartmann V., Khalid S.G., Hughes S., Zheng D. (2020). Comparison of different modulations of photoplethysmography in extracting respiratory rate: From a physiological perspective. Physiol. Meas..

[33] Khalid S.G., Ali S.M., Liu H., Qurashi A.G., Ali U. (2022). Photoplethysmography temporal marker-based machine learning classifier for anesthesia drug detection. Med. Biol. Eng. Comput..

[34] Amra B., Niknam N., Sadeghi M.M., Rabbani M., Fietze I., Penzel T. (2014). Obstructive sleep apnea and postoperative complications in patients undergoing coronary artery bypass graft surgery: A need for preventive strategies. Int. J. Prev. Med..

[35] Matyal R., Qureshi N.Q., Mufarrih S.H., Sharkey A., Bose R., Chu L.M., Liu D.C., Senthilnathan V., Mahmood F., Khabbaz K.R. (2021). Update: Gender differences in CABG outcomes-Have we bridged the gap?. PLoS ONE.

