# Supplementary material for: Predictors of Nocturnal Hypoxemic Burden in Patients Undergoing Elective Coronary Artery Bypass Grafting Surgery
Source: Biomedicines. 2023 Sep 28;11(10):2665. doi: 10.3390/biomedicines11102665 (PMC10603934; doi:10.3390/biomedicines11102665)
Supplement: Supplementary file 1 [file biomedicines-11-02665-s001.zip › biomedicines-2607601-supplementary.pdf]

Predictors of Nocturnal Hypoxemic Burden in Patients Undergoing Elective Coronary Bypass Grafting Surgery—Online data supplement

Supplementary tables

Table S1. Baseline variables of the sub-analysis population and patients excluded from the sub-analysis or had insufficient data on nocturnal hypoxemia (drop-out population). Data are presented as absolute and relative frequencies or mean ± standard deviation.

	**overall**	**sub-analysis population**	**drop-out population**	**p-value**
n (%)	600 (100)	429 (100)	171 (100)	
Age, years	66.6 ± 8.6	66.6 ± 8.5	66.7 ± 9.1	0.827 [T]
Male sex, n (%)	516 (86)	368 (86)	148 (86)	0.806 [Chi]
Body mass index, kg/m²	28.8 ± 4.8	28.6 ± 4.4	29.4 ± 6.0	0.071 [T]
Impaired LV ejection fraction <55%	115 (30)	98 (29)	17 (33)	0.614 [Chi]

NT-pro BNP: N-terminal pro-brain natriuretic peptide. [T] Student's t-test; [Chi] Chi-square test.

Table S2. Baseline variables of the sub-analysis population and patients excluded from the sub-analysis or had insufficient data on nocturnal hypoxemia. Data are presented as absolute and relative frequencies or mean ± standard deviation.

	**overall**	**sub-analysis population**	**withdrawal population**	**patients with insufficient data on nocturnal hypoxemia**	**p-value**
n (%)	600 (100)	429 (100)	101 (100)	70 (100)	
Age, years	66.6 ± 8.6	66.6 ± 8.5	66.6 ± 9.7	66.9 ± 8.2	0.951 [A]
Male sex, n (%)	516 (86)	368 (86)	89 (88)	59 (84)	0.754 [Chi]
Body mass index, kg/m²	28.8 ± 4.8	28.6 ± 4.4	30.1 ± 7.0	28.7 ± 4.9	0.052 [A]
Impaired LV ejection fraction <55%	115 (30)	98 (29)	7 (35)	10 (31)	0.845 [Chi]

NT-pro BNP: N-terminal pro-brain natriuretic peptide. Chi-square test; [A] ANOVA test.

Table S3. Patient characteristics. Data are presented as mean ± standard deviation, median (interquartile range) or absolute and relative frequencies.

	**no heart failure, no mild to moderate COPD, no obesity, no SDB**	**heart failure**	**mild to moderate COPD**	**obesity**	**SDB**	**2/4 comorbidities**	**≥3/4 comorbidities**	**p-value**
**Demographics**								
n (%)	134 (31)	19 (4)	14 (3)	50 (12)	63 (15)	109 (25)	40 (9)	
Age, years	66.3 ± 8.7	63.9 ± 11.7	63.2 ± 9.5	65.4 ± 8.2	69.0 ± 8.2	66.9 ± 7.6	66.6 ± 7.8	**0.025 A**
Male sex, n (%)	112 (84)	17 (89)	13 (93)	41 (82)	56 (89)	93 (85)	36 (90)	0.808 Chi
Body mass index, kg/m²	26.0 ± 2.5	24.9 ± 3.0	26.7 ± 2.0	32.8 ± 2.7	26.3 ± 2.6	30.9 ± 4.5	32.0 ± 3.8	0.429 A
**Cardiovascular risk factors**								
Hypertension, n (%)	105 (78)	14 (74)	11 (79)	46 (92) #	45 (71)	97 (89) #	38 (95) #	**0.004 Chi**
Hypercholesterinaemia, n (%)	78 (58)	9 (47)	7 (50)	34 (68)	45 (71)	72 (66)	21 (52)	0.183 Chi
Diabetes mellitus, n (%)	29 (22)	7 (37)	3 (21)	23 (46) #	19 (30)	41 (38) #	20 (50) #	**0.004 Chi**
Obesity, n (%)	0 (0)	0 (0)	0 (0)	50 (100) #	0 (0)	73 (67) #	34 (85) #	**<0.001 Chi**
Smoking, n (%)	84 (63)	16 (84)	8 (57)	32 (64)	42 (67)	74 (68)	32 (80)	0.269 Chi
**Comorbidities**								
Heart failure*, n (%)	0 (0)	19 (100) #	0 (0)	0 (0)	0 (0)	38 (35) #	30 (75) #	**<0.001 Chi**
NYHA III/IV, n (%)	15 (11)	3 (16)	2 (14)	13 (26) #	6 (9)	37 (34) #	20 (50) #	**<0.001 Chi**
History of myocardial infarction, n (%)	29 (22)	8 (42)	4 (29)	14 (28)	22 (33)	33 (30)	13 (32)	0.479 Chi
Atrial fibrillation, n (%)	15 (11)	5 (26)	0 (0)	3 (6)	5 (8)	26 (24) #	6 (15)	**0.004 Chi**
History of TIA or stroke, n (%)	14 (10)	1 (5)	1 (7)	9 (18)	12 (19)	17 (16)	4 (10)	0.422 Chi
Mild to moderate chronic obstructive pulmonary disease, n (%)	0 (0)	0 (0)	14 (100) #	0 (0)	0 (0)	12 (11) #	18 (45) #	**<0.001 Chi**
Renal failure†, n (%)	18 (14)	4 (21)	4 (29)	12 (24)	7 (11)	34 (31) #	9 (22)	**0.015 Chi**
Anemia‡, n (%)	25 (19)	5 (26)	3 (21)	9 (18)	12 (19)	22 (20)	11 (27)	0.912 Chi
Depression, n (%)	8 (6)	0 (0)	0 (0)	2 (4)	3 (5)	2 (2)	4 (10)	0.322 Chi
**Echocardiography parameters**								

LV ejection fraction, %	59.5 ± 8.1	45.4 ± 9.9 #	59.2 ± 7.9	61.5 ± 7.6	55.0 ± 12.7	52.1 ± 12.4 #	45.5 ± 14.3 #	**<0.001** A
Impaired LV ejection fraction <55%, n (%)	7 (11)	0 (0)	0 (0)	2 (10)	3 (11)	10 (17)	9 (32)	0.056 Chi
Left atrial enlargement, n (%)	35 (35)	5 (56)	3 (27)	20 (49)	18 (37)	49 (59) #	24 (83) #	**<0.001** Chi
**Laboratory data**								
NT-proBNP, pg/ml	159 (70; 472)	1961 (938; 3153) #	153 (88; 257)	153 (79; 409)	254 (94; 477)	453 (128; 1619) #	1924 (996; 2782) #	**<0.001** KW
Hemoglobin, g/dl	14.2 (13.3; 15.0)	14.3 (11.2; 15.2)	13.8 (12.0; 15.3)	14.5 (13.0; 15.3)	14.2 (13.4; 14.8)	13.9 (12.9; 14.6)	14.2 (12.7; 15.9)	0.805 KW
Hb1Ac, g/dl	5.7 (5.5; 6.2)	5.8 (5.3; 6.3)	5.8 (5.4; 7.4)	6.1 (5.6; 6.6)	5.8 (5.5; 6.4)	5.9 (5.5; 6.7)	6.1 (5.7; 6.9)	0.088 KW
Creatinine, mg/dl	0.95 (0.81; 1.05)	0.89 (0.81; 1.12)	0.97 (0.85; 1.24)	0.94 (0.82; 1.09)	0.94 (0.84; 1.07)	1.00 (0.85; 1.22)	1.00 (0.88; 1.18)	0.198 KW
GFR, ml/min/1,73 m^2	81 (71; 91)	89 (55; 91)	91 (53; 94)	82 (65; 92)	81 (68; 89)	76 (55; 89)	74 (61; 90)	0.132 KW
**Preoperative information on surgical treatment**								
CABG and valve surgery, n (%)	29 (22)	8 (42)	1 (7)	14 (28)	4 (6) #	27 (25)	12 (30)	**0.006** Chi
Number of coronary stenoses, n	3 (3; 4)	4 (3; 6)	4 (3; 5)	4 (3; 5)	4 (3; 5)	3 (3; 5)	3 (3; 5)	0.173 KW
Number of grafts, n	2 (2; 3)	2 (2; 3)	2 (2; 3)	3 (1; 3)	3 (2; 3)	2 (2; 3)	3 (2; 3)	0.514 KW

A ANOVA test; Chi Chi-square test; KW Kruskal-Wallis-test. NYHA: New York Heart Association; TIA: transient ischemic attack; LV: left ventricular; NT-proBNP: N-terminal pro-brain natriuretic peptide; HbA1c: glycosylated Hemoglobin, Type A1C; GFR: glomerular filtration rate; CABG: coronary artery bypass grafting. * n=363; NT-proBNP ≥450 pg/mL (patients <50 years of age), ≥900 pg/mL (patients ≥50 and <75 years of age) or ≥1800 pg/mL (patients ≥75 years of age); † glomerular filtration rate <60 ml/min/1.73 m^2; ‡ hemoglobin <12 g/dl (women) or hemoglobin <13 g/dl (men); # post hoc analysis <0.05, compared to patient group with no heart failure, no mild to moderate COPD, no obesity, no SDB.

Table S4. Nocturnal respiration data of the study population of patients (n = 429) without and with an elevated nocturnal hypoxemic burden index. Data are presented as median (interquartile range) or absolute and relative frequencies.

	**no heart failure, no mild to moderate COPD, no obesity, no SDB**	**heart failure**	**mild to moderate COPD**	**obesity**	**SDB**	**2/4 comorbidities**	**≥3/4 comorbidities**	**p-value**
Total recording time, min	487 (471; 501)	484 (453; 494)	477 (465; 486)	484 (466; 501)	491 (465; 507)	485 (463; 507)	488 (471; 504)	0.508 KW
Apnea hypopnea index, per hour	6.5 (3.7; 10.7)	7.9 (4.6; 11.3)	5.4 (3.4; 11.2)	9.7 (6.5; 11.6)	22.0 (17.9; 34.7)	23.9 (19.0; 33.4)	28.7 (21.3; 43.7)	**<0.001** KW
Obstructive apnea index, per hour	1.2 (0.4; 2.2)	0.8 (0.1; 1.8)	1.0 (0.5; 1.8)	1.1 (0.5; 2.1)	4.9 (2.4; 11.5)	4.0 (1.5; 9.2)	4.9 (1.8; 12.0)	**<0.001** KW
Central apnea index, per hour	0.9 (0.3; 2.2)	1.4 (0.1; 2.5)	1.6 (0.4; 3.4)	0.8 (0.2; 2.8)	6.2 (2.4; 13.7)	5.7 (1.8; 13.1)	4.9 (0.4; 23.3)	**<0.001** KW
Oxygen desaturation index, per hour	4.8 (1.9; 8.4)	7.4 (3.1; 11.2)	4.5 (2.1; 10.5)	7.4 (5.4; 9.4)	17.8 (14.5; 29.8)	21.3 (15.1; 30.8)	25.6 (17.0; 42.1)	**<0.001** KW
Mean SpO_2, %	93 (92; 94)	93 (92; 94)	90 (92; 93)	92 (91; 93)	93 (91; 94)	92 (90; 93)	91 (89; 92)	**<0.001** KW
Min SpO_2, %	85 (82; 88)	85 (83; 88)	83 (81; 86)	82 (79; 85)	82 (78; 84)	80 (75; 84)	76 (73; 83)	**<0.001** KW
T90/TRT, %	1.6 (0.2; 11.3)	2.4 (0.2; 10.6)	5.0 (2.3; 35.9)	7.6 (2.4; 20.1)	9.9 (2.4; 23.9)	12.6 (5.5; 31.2)	34.8 (10.8; 65.5)	**<0.001** KW
$T90_{desaturation}$/TRT, %	0.6 (0.1; 2.6)	0.6 (0.1; 3.6)	1.4 (0.6; 6.2)	1.6 (0.7; 4.8)	6.3 (1.5; 11.9)	7.6 (3.2; 14.4)	13.6 (7.7; 28.4)	**<0.001** KW
$T90_{non-specific}$/TRT, %	0.4 (0.0; 7.5)	0.9 (0.0; 6.6)	4.1 (0.9; 30.7)	6.8 (0.7; 18.9)	1.4 (0.1; 10.0)	3.7 (0.3; 14.8)	16.7 (1.4; 39.4)	**<0.001** KW
Sleep-disordered breathing (Apnea-hypopnea index ≥15/hour), n (%)	0 (0)	0 (0)	0 (0)	0 (0)	63 (100)	95 (87)	40 (100)	**<0.001** Chi
Obstructive sleep apnea (Apnea-hypopnea index ≥15/hour), n (%)	0 (0)	0 (0)	0 (0)	0 (0)	27 (43)	37 (34)	18 (45)	
Central sleep apnea (Apnea-hypopnea index ≥15/hour), n (%)	0 (0)	0 (0)	0 (0)	0 (0)	36 (57)	58 (53)	22 (55)	

Chi Chi-square test; KW Kruskal-Wallis-test. TRT: artefact-free total recording time.

Supplementary figure

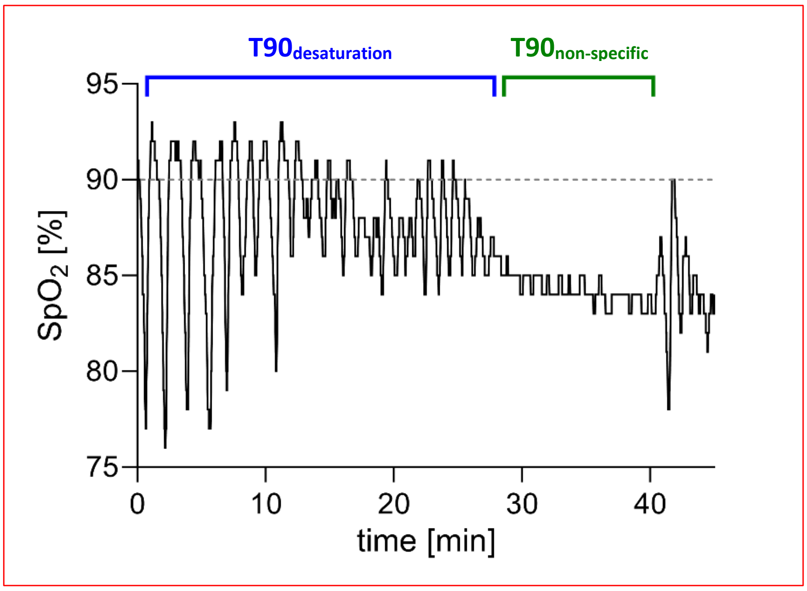

Figure S1. Descriptive diagram of T90 with acute oxygen desaturation events accompanied by resaturation ($T90_{desaturation}$) and T90 associated with non-specific drifts in SpO2 or incomplete resaturation ($T90_{non\text{-}specific}$) as obtained from raw oximetry data with the use of a custom MATLAB software algorithm.

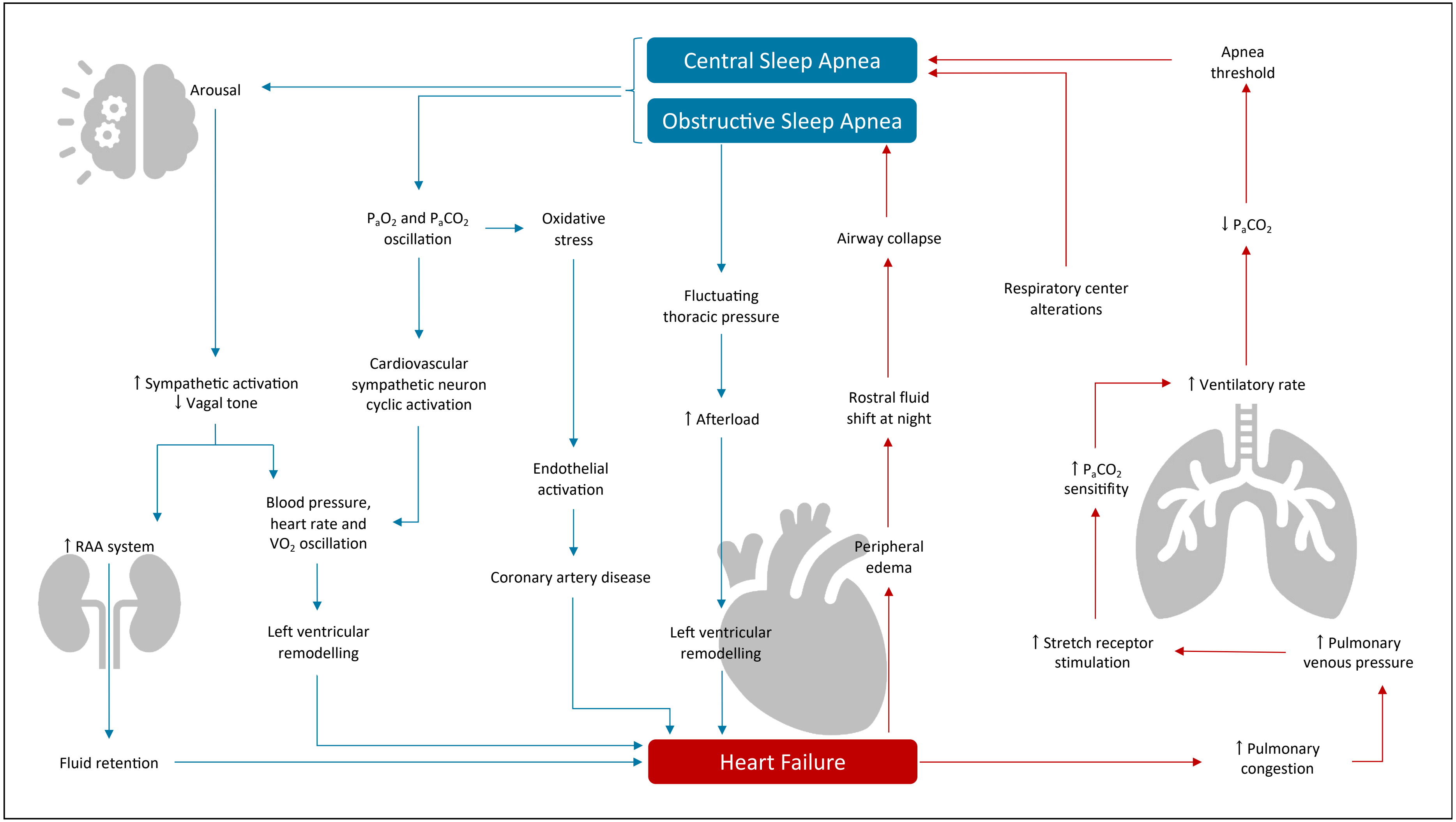

Figure S2. Schematic model depicting the relationship between sleep apnea and heart failure (illustration modified from Parati G et al. 2016)[2]. RAA: Renin-angiotensin-aldosterone; VO_2: oxygen consumption.
